# The Role of Decay Accelerating Factor in Environmentally Induced and Idiopathic Systemic Autoimmune Disease

**DOI:** 10.1155/2014/452853

**Published:** 2014-01-27

**Authors:** Christopher B. Toomey, David M. Cauvi, Kenneth M. Pollard

**Affiliations:** ^1^Department of Ophthalmology, Duke University School of Medicine, Albert Eye Research Institute, Durham, NC 27710, USA; ^2^Department of Surgery and Center for Investigations of Health and Education Disparities, University of California, San Diego, La Jolla, CA 92037, USA; ^3^Department of Molecular and Experimental Medicine, The Scripps Research Institute, La Jolla, CA 92037, USA

## Abstract

Decay accelerating factor (DAF) plays a complex role in the immune system through complement-dependent and -independent regulation of innate and adaptive immunity. Over the past five years there has been accumulating evidence for a significant role of DAF in negatively regulating adaptive T-cell responses and autoimmunity in both humans and experimental models. This review discusses the relationship between DAF and the complement system and highlights major advances in our understanding of the biology of DAF in human disease, particularly systemic lupus erythematosus. The role of DAF in regulation of idiopathic and environmentally induced systemic autoimmunity is discussed including studies showing that reduction or absence of DAF is associated with autoimmunity. In contrast, DAF-mediated T cell activation leads to cytokine expression consistent with T regulatory cells. This is supported by studies showing that interaction between DAF and its molecular partner, CD97, modifies expression of autoimmunity promoting cytokines. These observations are used to develop a hypothetical model to explain how DAF expression may impact T cell differentiation via interaction with CD97 leading to T regulatory cells, increased production of IL-10, and immune tolerance.

## 1. Introduction

Decay accelerating factor (DAF) was first described in 1969 in human erythrocytes that inhibited complement activation *in vitro* [1]. (The gene and protein designations used for decay accelerating factor in this paper are *DAF* for the human gene and DAF for the human protein. The mouse genes are *Daf1* and *Daf2* and the protein is DAF1.) However, its biological significance was not appreciated until 1982 when the human protein was isolated and deficiency of DAF was found in patients with paroxysmal nocturnal hemoglobinuria (PNH) [1–3]. The major function of DAF is to protect self-cells from complement-mediated attack by inhibiting the cleavage of C3 and C5, blocking the formation of C3 and C5 convertases, and accelerating their decay [4]. In humans, DAF is expressed as a posttranslationally modified glycosylphosphatidylinositol- (GPI-) anchored molecule [5, 6]. In mice, functionally equivalent, GPI-anchored, and transmembrane-anchored DAF proteins are produced, which are derived from two different genes, *Daf1* and *Daf2*, respectively [7]. *Daf1* is ubiquitously expressed, whereas, *Daf2* is mostly present in the testis and splenic dendritic cells [8]. DAF is also found in soluble form in plasma, cerebrospinal fluid, saliva, synovial fluid, and urine [9]. In humans, *DAF* is encoded by a single gene which maps to q32 on chromosome 1 [10]. It is widely expressed on the surface of all major circulating blood cells as well as epithelial and endothelial cells [9, 11]. Constitutive expression can vary depending on tissue and cell type [8, 12]. In human cells, *DAF* expression is modulated by cytokines such as IL-1, IL-6, TNF-*α*, TGF-*β*1, and IFN-*γ*, prostaglandins, and tissue-specific factors and controlled by the transcription factor SP1 [13–17]. In 1988, DAF was found to be rapidly expressed in T-cells activated by mitogens and *in vitro* stimulation with anti-DAF antibodies led to phosphatidylinositol-specific phospholipase C dependent T-cell proliferation [18]. This led to the hypothesis that an alternative function of DAF may be to regulate T-cell tolerance. Subsequently, DAF has been shown to negatively regulate a variety of autoimmune diseases including animal models of antiglomerular basement membrane glomerulonephritis, experimental autoimmune myasthenia gravis (EAMG), experimental autoimmune encephalomyelitis (EAE), cardiac allograft rejection, and idiopathic and induced models of systemic lupus erythematosus (SLE) [19–24].

## 2. Complement System and DAF

**Figure 1:**
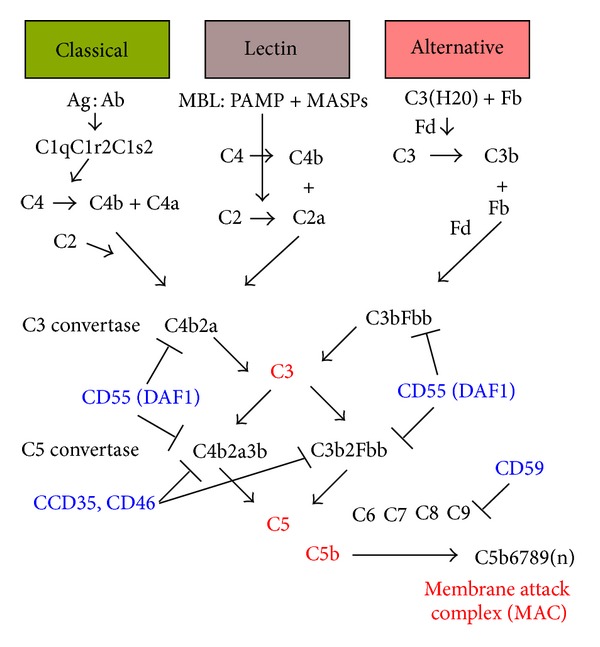
Overview of the complement system. Stimulated by antigen : antibody complexes, bacterial cell surfaces, and spontaneous hydrolysis, respectively, the classical, lectin, and alternative pathways converge to convert C3 to C3 convertase, an enzyme capable of initiating a cascade that results in cell membrane pore formation and subsequent cell lysis known as the membrane attack complex (MAC). To protect host cells from complement activation four main plasma membrane complement regulatory proteins are expressed, CD59 (membrane inhibitor of reactive lysis (MIRL)), CD35 (type 1 complement receptor (CR1)), CD46 (membrane cofactor protein, (MCP)), and CD55 (decay accelerating factor (DAF)), that interrupt the complement cascade on self-cells. CD59 blocks MAC complex formation, CD35 acts as a cofactor to inactivate C3b and C4b by factor I, and interacts with C3b and C4b to promote immune-complex removal, CD46 acts as a cofactor to inactivate C3b and C4b through factor I and DAF inhibits the cleavage of C3 and C5 by blocking the formation of C3 and C5 convertases and accelerating their decay. MBL: mannose-binding lectin; MASPs: MBL-associated serine proteases; PAMP: pathogen-associated molecular pattern; Fb: factor B.

The complement system is among the oldest evolutionary components of the immune system. It was discovered in 1896 as a heat-labile fraction of serum that led to opsonization of bacteria. Biochemical characterization showed that the complement system is composed of over 30 proteins that function to mediate removal of apoptotic cells and eliminate pathogens. Three separate pathways (i.e., classical, alternative, and lectin pathways) converge to convert C3 to C3 convertase, an enzyme capable of initiating a cascade that results in cell membrane pore formation and subsequent cell lysis known as the membrane attack complex (MAC) (Figure 1). To protect host cells from complement activation four plasma membrane complement regulatory proteins are expressed, CD59 (membrane inhibitor of reactive lysis (MIRL)), CD35 (type 1 complement receptor (CR1)), CD46 (membrane cofactor protein (MCP)), and CD55 (decay accelerating factor (DAF)), that interrupt the complement cascade on self-cells. CD59 blocks MAC complex formation [25], CD35 acts as a cofactor to inactivate C3b and C4b by factor I and interacts with C3b and C4b to promote immune-complex removal [9], and CD46 acts as a cofactor to inactivate C3b and C4b through factor I [9]. DAF inhibits the cleavage of C3 and C5 by blocking the formation of C3 and C5 convertases and accelerating their decay [4]. The original premise of the complement system as a member of the innate immune system, however, was redefined three decades ago when it was shown that complement participates in B- and T-cell responses especially the induction and regulation of type I helper (T_h_1) CD4^+^ T-cell responses [26].

## 3. DAF in Human Autoimmune Diseases

Deficiency of DAF is found in PNH patients, a GPI linked protein deficiency, which leads to systemic complications particularly through intravascular hemolysis and platelet activation [27–30]. Interestingly, however, it has been reported that DAF (CD55) deletion, also known as the Inab phenotype, hardly alters the sensitivity of cells to lysis by complement and only with inhibition of CD59 does hemolysis occur *in vitro* [31–34]. Deficient expression of the complement regulatory proteins CD55 and CD59 has been found in a variety of human diseases (Table 1) [35–61], however, most prevalently in autoimmune hemocytopenia, autoimmune vasculitis, and other diseases involving dysregulated immune responses [41, 44, 62–68]. It is important to note that deficient expression seen in these diseases does not always mimic the PNH pattern of deficiency on specific cell populations but instead shows decreased expression of all cells, suggesting the nonclonal nature of the deficiency [44].

A paradox exists in SLE where complement activation is associated with tissue injury, yet deficiencies in the early classical complement component pathways predispose to SLE [69, 70]. C1q deficiency due to either gene deletion or anti-C1q autoantibodies has been shown to be related to disease activity; in fact between 53 and 93% of SLE patients have been reported to have low C1q during active disease [71]. This has been reconciled by studies demonstrating the protective role of classical complement pathway components (C1, C2, and C4) in facilitating the clearance of immune complexes as well as autoantigens in apoptotic debris [70, 72]. However, decreased complement regulatory components on lymphocytes and erythrocytes, including CD55 and CD59, have been shown to be associated with lymphopenia and autoimmune hemolytic anemia (AHIA). In 2003, it was shown that AIHA patients with SLE had decreased levels of DAF compared to SLE patients without AIHA but similar levels to patients with AIHA but without SLE [73]. No correlation was seen, however, between IgG or IgM antiphospholipid antibodies [73]. In a follow-up study an analysis of 40 SLE patients with and without lymphopenia showed that mean fluorescence intensities (MFI) of CD55 and CD59 were diminished on T- and B-cells in lymphopenic patients compared to nonlymphopenic patients [41]. These results were unrelated to disease activity [41]. In two recent studies Alegretti et al. conducted a peripheral blood flow cytometric analysis of SLE patients and healthy controls [67, 68]. They found a decrease in the percent of DAF high peripheral lymphocytes and decreased DAF MFI in peripheral lymphocytes in patients with lymphopenia; however, no difference was seen in non-lymphopenic SLE patients compared to controls [67, 68]. Mean DAF levels were also shown to be decreased on red blood cells and granulocytes but not on monocytes; however, no relationship between disease activity and DAF lymphocyte levels was seen [67, 68]. Collectively, these studies suggest a role for complement regulatory proteins in the pathophysiology of a subset of SLE patients with lymphopenia and AIHA. The most plausible explanation is antibody-dependent cellular cytotoxicity and complement-mediated cell lysis which would explain the correlation between reduced CD55 and CD59 in AIHA and lymphopenic SLE populations. An alternative hypothesis that decreased CD55 lymphocyte levels predispose a subset of SLE patients to lymphopenia should not be ignored.

Not nearly as many studies have investigated the relationship between DAF and other systemic autoimmune diseases. One study found that percentages of DAF negative CD4^+^ and CD8^+^ T-cell subsets were higher in Sjogren's Syndrome (SS) patients [36]. The DAF expression observed in other circulating blood cells was not changed [36]. However, these results were not thought to be due to increases in DAF low cells but rather a decrease in DAF high cells [36]. In patients with systemic sclerosis (Scleroderma or SSc), DAF was found to be decreased or undetectable in endothelium of both lesional skin and nonlesional skin compared to controls [37]. Interestingly this result was later reproduced in morphea lesions, suggesting a link between systemic autoimmune disease skin lesions and low DAF levels, although low DAF levels are also seen in psoriatic skin lesions [38, 39]. These studies, however, are limited by their power and lack of supporting follow-up studies.

## 4. DAF in Animal Models of Systemic Autoimmune Disease

DAF1 has been shown to play a role in the maintenance of immune tolerance in mouse models of autoimmune disease. Deletion of *Daf1* was shown to increase susceptibility to antiglomerular basement membrane disease and to markedly enhance susceptibility in a mouse model of myasthenia gravis [20, 23]. This increased susceptibility was later shown to result from the influence of DAF1 on T-cell hypersensitivity, when it was demonstrated that *Daf1* ^*−/−*^ T-cells displayed a C3 dependent enhanced response to antigen restimulation resulting in increased IFN-*γ* [24, 74, 75]. Similar results were found in MRL-*Fas* ^*lpr*^ mice, an idiopathic SLE model, where deletion of *Daf1* resulted in exacerbated lymphadenopathy and splenomegaly, increased serum antichromatin autoantibody, and aggravated dermatitis [21]. In a follow-up study it was shown that, aside from local skin inflammation, these effects were largely complement independent [76].

These observations stimulated us to investigate the role of DAF1 in murine mercury-induced autoimmunity (mHgIA). Mice exposed to mercury develop lymphadenopathy, hypergammaglobulinemia, humoral autoimmunity, and immune-complex disease, which are consistent with the systemic features observed in SLE [77, 78]. All forms of inorganic mercury tested, including HgCl_2_, vapor, or dental amalgam, elicit the same disease [79–81] as do different routes of administration [82, 83]. Sensitivity to mHgIA is influenced by both MHC and non-MHC genes and covers the spectrum from nonresponsiveness to overt systemic autoimmunity [82, 84–86]. Disease expression is influenced by TCR costimulatory molecules including CD28 and CD40L [87, 88], proinflammatory cytokines, including IFN-*γ* [80, 89] and IL-6 [90], and modulators of innate immunity, including endosomal Toll-like receptors (TLR) [91, 92], demonstrating that multiple checkpoints and pathways are implicated in the regulation of the disease. In addition lupus prone strains exhibit accelerated and more severe systemic autoimmunity following mercury exposure [93–95]. Environmental factors have been associated with systemic autoimmune diseases in humans [96, 97]. Exposure to mercury has been implicated as an environmental trigger in the induction of autoimmunity [98–100] including production of autoantibodies and proinflammatory cytokines, such as IL-1*β*, TNF-*α*, and IFN-*γ* [101], and membranous nephropathy [102].

We found that autoimmune-prone NZB mice had low endogenous levels of DAF1 while mHgIA-resistant DBA/2 animals have high endogenous levels [103]. Furthermore, we showed that induction of mHgIA in B10.S mice was associated with reduction of DAF1 on activated CD4^+^ T cells [103]. Both these observations, reduction of DAF1 in autoimmune-prone mice and reduction upon induction of mHgIA, support the argument that DAF1 is required to maintain immune tolerance. In a follow-up study the absence of *Daf1* in C57BL/6 mice was shown to cause increased serum autoantibodies and exacerbated hypergammaglobulemia following mercury exposure (Table 2) [19]. This response, however, could not be explained by increased T-cell activation but rather was explained by increased levels of IFN-*γ*, IL-2, IL-4, and IL-10 (Table 2) [19]. Furthermore, depletion of C3 was found to have no major effects on development of mHgIA suggesting that the role of DAF1 in mHgIA is independent of an intact complement system [104]. This is supported by results showing that C3 levels are not affected following mercury exposure of mHgIA sensitive or resistant strains (Pollard, unpublished results). Thus our findings and those of others [76] suggest that DAF1 may regulate idiopathic and induced models of systemic autoimmunity in a complement-independent fashion.

## 5. How Does DAF1 Regulate Immune Tolerance?

Several models of systemic autoimmunity exhibit disease independent of an intact complement system, yet an exacerbated phenotype is observed by DAF1 deletion [19, 21, 76, 104, 105]. This suggests that complement-independent effects of DAF1 are the major contributors to tolerance induction via the interaction of DAF (CD55) with its natural ligand, CD97. CD97 is a member of the epidermal growth factor-like, seven span transmembrane (EGF-TM7) family of proteins, is expressed on macrophages, granulocytes, dendritic cells, and smooth muscle cells, and is rapidly upregulated on activated T- and B-cells [106, 107]. In the mouse alternative RNA splicing produces two isoforms with three or four epidermal growth factor-like (EGF) domains (namely CD97(EGF1,2,4) and CD97(EGF1,2,3,4)) and a third isoform with a protein module inserted between EGF domains 2 and 3 (namely, CD97(EGF1,2,X,3,4)) [108]. DAF (CD55) binds to both CD97(EGF1,2,4) and CD97(EGF1,2,3,4) but not to CD97(EGF1,2,X,3,4) [108].

Structural studies of a model of the CD55-CD97 complex reveal that the sites of interaction of CD97 and complement components occur on opposite faces of CD55 arguing that CD55 can bind to CD97 and complement independently [106]. Activation of human CD4^+^ T cells in the presence of anti-CD3 and recombinant CD97 (rCD97) results in increased IL-10 production that is IL-2 dependent [109]. Costimulation of naïve human CD4^+^ T-cells via interaction of CD97/CD55 leads to T regulatory type 1 (Tr1) activation, expansion, and function [110]. Antibody mediated blockade of CD97-CD55 interaction inhibits proliferation and IFN-*γ* production [106]. These properties of CD97-CD55 co-stimulation, particularly IL-2 dependent IL-10, are consistent with stimulation of human CD4^+^ T cells by another complement regulatory protein, CD46. Activation via CD3/CD46 induces a T_h_1 phenotype with significant IFN-*γ* production [111]. However in the presence of IL-2, immunoregulatory IL-10 is produced resulting in a T regulatory phenotype [111, 112] capable of suppressing antigen-specific T cells [113]. We hypothesize that the immunosuppressive potential of CD55 [24, 75] lies in its interaction with CD97 leading to a T regulatory cell phenotype under appropriate conditions of increasing IL-2, as would happen in an inflammatory response [114]. Thus, like CD46 activation, CD97 mediated activation of CD55 acts to switch T_h_1 effector CD4^+^ T cells toward IL-10 producing immunosuppressive cells [112]. We have shown that activation of murine CD4^+^ T cells with anti-CD3 and rCD97 leads to a cytokine profile with increased IL-10 and reduced IL-17 and IL-21 compared to a more proinflammatory profile of elevated IFN-*γ*, IL-2, IL-4, IL-10, IL-17, and IL-21 elicited by conventional costimulation by anti-CD3/anti-CD28 [19]. Increased IL-10 and reduced IFN-*γ* and IL-17 suggest that rCD97 may be driving T cells to differentiate into T regulatory cells rather than autoimmune promoting T_h_1 and T_h_17 cells, but it remains to be determined whether rCD97 activated CD4^+^ T cells have immunoregulatory activity.

**Figure 2:**
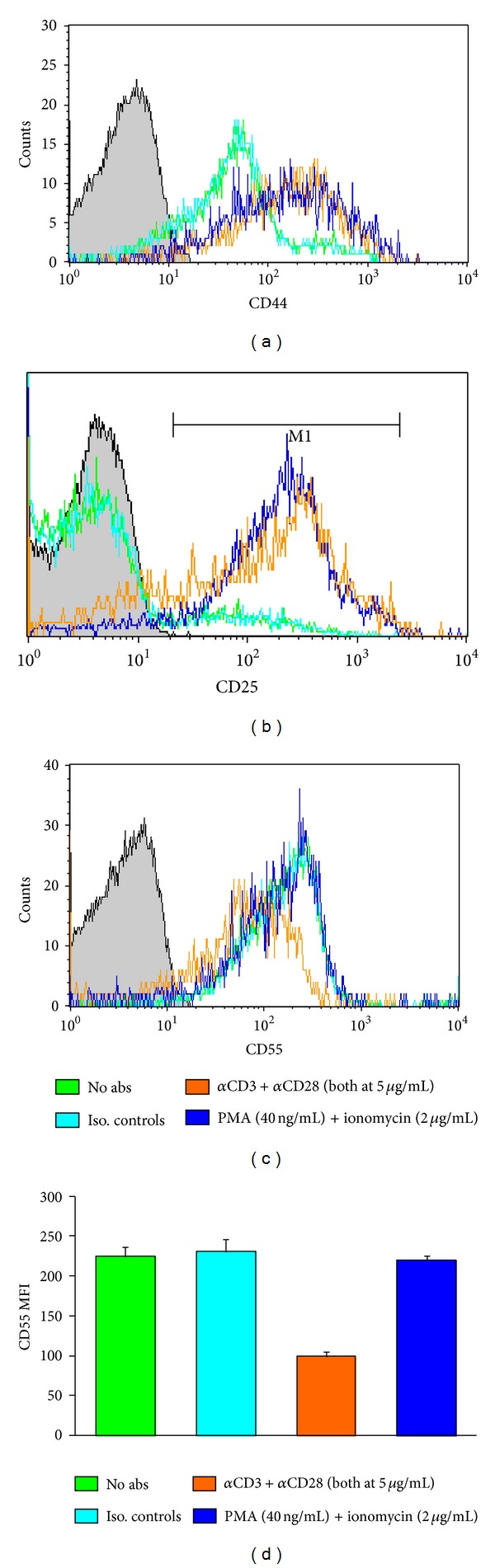
Anti-CD3/anti-CD28 but not phorbol 12-myristate 13-acetate (PMA)/ionomycin lowers CD55 expression of CD4^+^ T-cells. Lymph node cells from B10.S mice were cultured with no additives (green), Ig isotype controls (light blue), anti-CD3/anti-CD28 (5 *μ*g/mL each) (orange), or phorbol 12-myristate 13-acetate (PMA) (40 ng/mL) and ionomycin (2 *μ*g/mL) (dark blue). Cells were incubated at 37°C and 5% CO_2_ for 72 hr and then analyzed by flow cytometry to determine activation status (CD44^hi^, CD25^hi^) and then CD55 expression of activated cells. *n* = 4/group.

Our previous studies had established that activated CD4^+^ T cells have reduced CD55 expression [103, 115]. However it was unclear if conventional activation of CD4^+^ T cells with anti-CD3/anti-CD28 could mimic the *in vivo* reduction of CD55 in idiopathic SLE or mHgIA [103]. Experimental comparison between anti-CD3/anti-CD28 and PMA/ionomycin activation (Figure 2) revealed that anti-CD3/anti-CD28 activation does indeed reduce CD55 expression of CD4^+^ T-cells by about 50% in B10.S mice which is very similar to the reduction found in mercury-exposed B10.S mice and constitutively in autoimmune prone NZB mice [103]. Unexpectedly, PMA/ionomycin activation of CD4^+^ T cells did not affect CD55 expression (Figure 2) indicating that direct intracellular signaling of protein kinase C (PKC) does not impact CD55 expression and that CD55 reduction is mediated by events occurring at the cell surface. Whether CD4^+^ T cell activation in the presence of anti-CD3/rCD97 also reduces CD55 remains to be determined.

**Figure 3:**
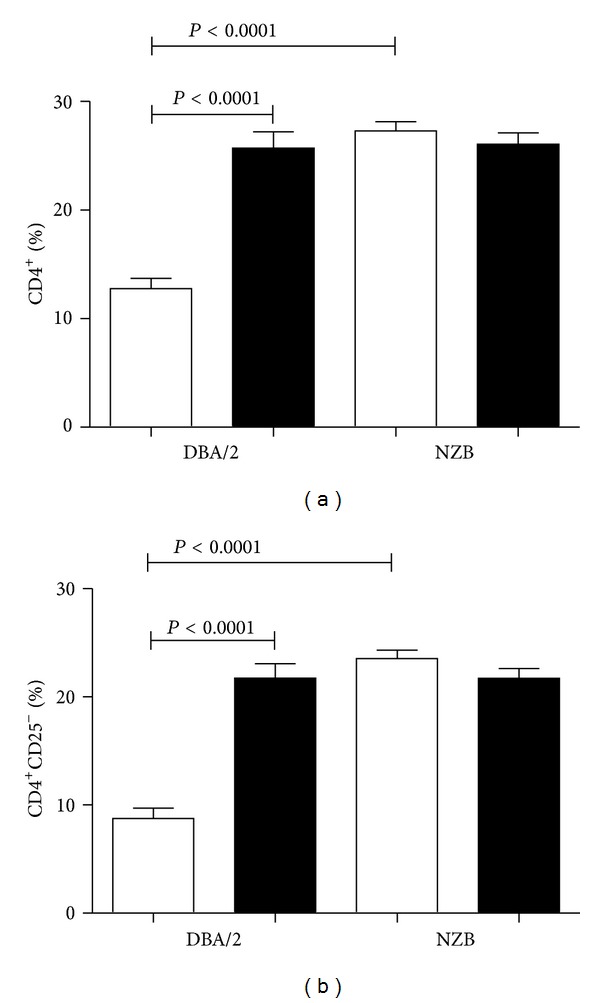
CD4^+^ T-cell expansion in mHgIA-resistant DBA/2 consists of CD25 negative cells. NZB and DBA/2 mice were exposed to PBS (white bar) or HgCl_2_ (black bar) for 5 weeks. Splenocytes were then cultured in the presence of PMA/ionomycin and analyzed for percent CD4^+^ T cells (a) and CD4^+^CD25^−^ T cells (b). Percentages represent cells in total spleen cells. *n* = 4/group.

**Figure 4:**
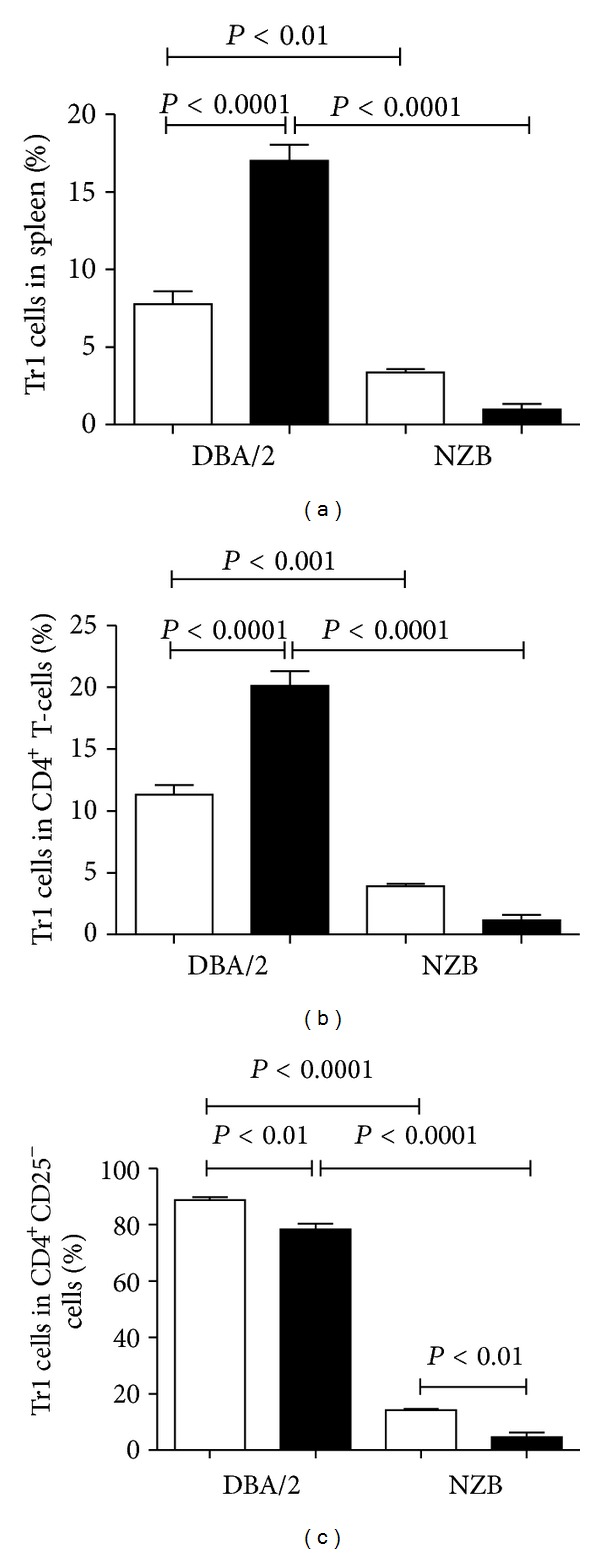
CD4^+^CD25^−^ T cells in mHgIA-resistant DBA/2 possess the cytokine phenotype of regulatory T-cells. NZB and DBA/2 mice were exposed to PBS (white bar) or HgCl_2_ (black bar) for 5 weeks. Splenocytes were then cultured in the presence of PMA/ionomycin and CD4^+^CD25^−^ T-cells analyzed for the cytokine phenotype of IL-10^+^IL-4^−^ (Tr1 cells). (a) shows the percent of Tr1 cells in total spleen. (b) shows the percent of Tr1 cells in CD4^+^ cells. (c) shows the percent of Tr1 cells in CD4^+^CD25^−^ cells. *n* = 4/group.

The increased expression of IL-10 in human and murine CD4^+^ T cells stimulated by anti-CD3 and rCD97 [19, 109] suggests a regulatory T-cell phenotype [110, 116, 117]. As CD55 is required for this response, we asked whether a difference in CD55 expression might affect the generation of regulatory T cells. NZB and DBA/2 mice have reduced and elevated CD55, respectively, and exposure to HgCl_2_ exacerbates autoimmunity in NZB while DBA/2 mice are resistant [103]. Thus regulatory T cells may be more common in DBA/2 than NZB mice. To examine this possibility NZB and DBA/2 mice were exposed to HgCl_2_ and splenocytes were then cultured *in vitro* in the presence of PMA/ionomycin and analyzed for the presence of markers of T regulatory type 1 (Tr1) (CD4^+^CD25^−^IL-10^+^IL-4^−^) cells [118]. This protocol increased CD4^+^ T cells in HgCl_2_ exposed DBA/2 mice but not in NZB mice (Figure 3(a)). This was an unexpected finding given that DBA/2 mice are resistant to mHgIA, however these cells were primarily of the CD4^+^CD25^−^ type (Figure 3(b)) indicating that they were not conventionally activated CD4^+^ T cells which can express CD25 [103]. CD4^+^CD25^−^ T cells were then examined for expression of both IL-10 and IL-4 to identify Tr1-like cells (Figure 4). In total spleen, Tr1-like cells were dramatically increased following HgCl_2_ in DBA/2 mice but were reduced in NZB mice (Figure 4(a)). A similar situation was found when only CD4^+^ cells in the spleen were analyzed (Figure 4(b)). Finally we asked what percentage of CD4^+^CD25^−^ cells were IL-10^+^IL-4^−^ and found that the vast majority in the DBA/2 mice possessed the cytokine phenotype of T regulatory cells while such cells were much fewer in number in the NZB mice (Figure 4(c)). The greater percentage of putative Tr1 cells in PBS treated DBA/2 mice compared to NZB mice and the changing percentages of Tr1-like cells following mercury exposure are consistent with the sensitivity of these strains to mercury-induced autoimmunity. We hypothesize that the constitutively reduced CD55 expression in NZB mice [103] reduces CD55-CD97 interaction and the generation of Tr1 cells but favors T_h_1 responses. In contrast, DBA/2 mice, with a higher level of CD55 that is not impacted by mercury exposure [103], are able to maintain a higher level of CD55-CD97 interaction which favors regulatory T-cell generation and tolerance to mHgIA.

**Figure 5:**
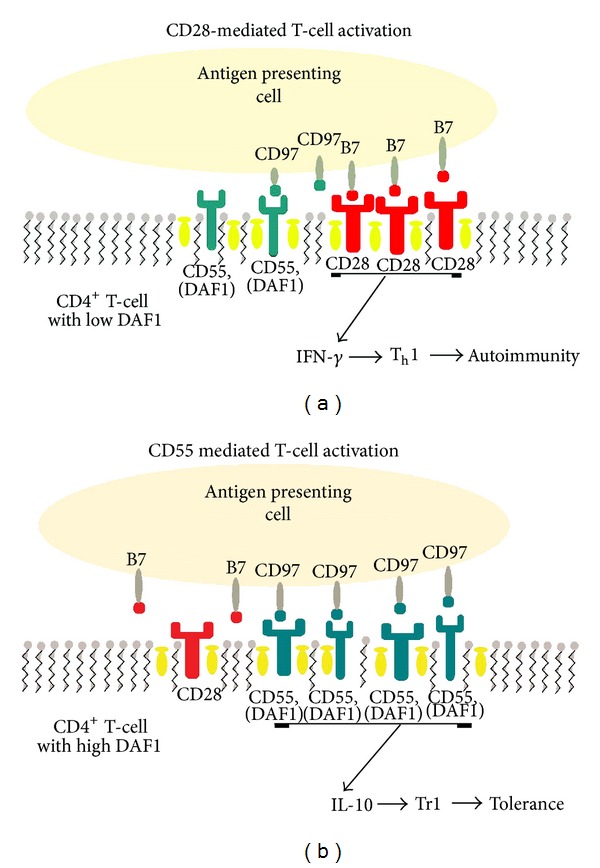
Hypothetical DAF function during T-cell costimulation. Conventional T-cell activation via CD28 cross-linking leads to the formation of lipid raft clusters which exclude CD55 (a) resulting in proinflammatory (i.e., IFN-*γ*) cytokine production which, under suitable conditions, may lead to T_h_1-mediated autoimmunity such as mHgIA. Conversely, increasing the surface density of DAF leads to less efficient CD28-mediated T cell activation (b) and in the presence of CD97 potentiates CD55 signaling leading to increased production of IL-10, a T regulatory cell phenotype and immune tolerance.

Our hypothesis is supported by studies comparing CD28 and CD55 mediated T cell activation. CD28 and GPI-anchored proteins, like DAF1, exist in detergent-resistant microdomains or lipid rafts, and their engagement leads to redistribution and clustering at the site of the T cell receptor (TCR) [119]. Moreover, CD28 cross-linking leads to the formation of lipid raft clusters which exclude CD55 and vice versa [120]. It has also been argued that recruitment and crosslinking of GPI-anchored proteins, such as CD55, are less efficient at T cell activation than that elicited by crosslinking of CD28 [119–121]. These observations raise the possibility that the cell surface density of DAF influences T cell activation by competing with other costimulatory molecules. Thus reduced levels of DAF favor more efficient T cell activation via CD28 crosslinking (Figure 5). Conversely increasing the surface density of DAF1 would affect the effectiveness of CD28-mediated T cell activation. This supports observations by us [19] and others [109] that preferential costimulation via CD28 elicits a different cytokine profile than that produced by costimulation via DAF1 (CD55).

## 6. Conclusion

DAF serves a complex role in the immune system through complement-dependent and -independent functions in the regulation of innate and adaptive immunity. Initial reports on DAF1 in systemic autoimmune disease models suggested that it played a role in regulating adaptive immune responses. In the context of mHgIA our lab has shown that (1) DAF1 is constitutively reduced in mice prone to systemic autoimmune disease, (2) mHgIA is associated with reduced DAF1 expression on T-cells, and (3) interaction of DAF1 with its natural ligand, CD97, can regulate cytokine expression. These results suggest that understanding how mercury exposure reduces DAF1 expression may lead to approaches to regulate DAF in SLE. Evidence in patients shows that decreased levels of DAF on lymphocytes are associated with lymphopenia in SLE as well as in T cells of SS and the endothelial skin lesions of SLE, SSc, and other vasculitic diseases. DAF1 has both complement-dependent and- independent effects by regulating T-cell pro-inflammatory cytokine production *in vivo*. In systemic autoimmune disease models, such as mHgIA, complement-independent effects of DAF1 appear to be the major contributors regulating disease. Based on our mHgIA studies, we hypothesize that CD55 : CD97 interaction at the immunological synapse can regulate CD28 crosslinking and promote a Tr1-like phenotype, either by direct CD55 signal transduction or possibly through blocking of CD28 costimulation, and expression of a Treg cytokine profile.

## Figures and Tables

**Table 1:** Decreased DAF expression in human disease.

Disease	Cell surface DAF deficiency	Citation
Immune dysregulation		
Sjogren's Syndrome	T-lymphocytes	[36]
SLE with lymphopenia and anemia	T-lymphocytes (CD8+), endothelium, lymphocytes, and anemia	[38, 41, 73]
Psoriatic skin	Epithelium and endothelium	[39]
Systemic sclerosis	Endothelium	[37]
Vasculitic skin lesions	Endothelium	[35]
Recurrent pregnancy loss in aPL	Endometrium	[42]
Autoimmune hemocytopenia	Platelets, lymphocytes, and RBC	[43, 44]
Rheumatoid arthritis	Neutrophils and RBC	[45, 46]
Myasthenia gravis	SNP with decreased expression	[47]
Vitiligo	Whole epidermis	[48]
Asthma	Bronchial epithelial cells and SNP	[49, 50]
Proliferative disorders		
Myelodysplastic syndrome	Granulocytes and RBC	[51, 52]
Plasma cell dyscrasias	RBC	[53]
Lymphoproliferative disorders	RBC	[54]
Anemias		
Anemia of malaria	RBC	[55–59]
Aplastic anemia	RBC	[51]
HIV	RBC, lymphocytes, and PBMC	[60, 61]

RBC: red blood cell; SNP: single-nucleotide polymorphism; aPL: antiphospholipid; PBMC: peripheral blood mononuclear cell.

**Table 2:** Effect of *Daf1* deletion on mHgIA*.

Serum autoantibodies	
ANA	↑↑↑
AntiChromatin abs	↑↑↑
Serum immunoglobulins	
IgG	↑
IgG1	NC
IgG2a	NC
T-cell activation	
CD4^+^CD44^high^	NC
Cytokines	
IFN-*γ*	↑
IL-4	↑
IL-2	↑
IL-10	↑
IL-17	NC
IL-22	NC
TGF-*β*	NC

*Data from Toomey et al., 2010 [19].

↑: *P* < 0.05; ↑↑↑: *P* < 0.0001; NC: no change.
